# *De novo* transcriptome sequencing of *Impatiens uliginosa* and the analysis of candidate genes related to spur development

**DOI:** 10.1186/s12870-022-03894-1

**Published:** 2022-12-01

**Authors:** Yang Li, Chun-Mei Wei, Xin-Yi Li, Dan-Chen Meng, Zhi-Jia Gu, Su-Ping Qu, Mei-Juan Huang, Hai-Quan Huang

**Affiliations:** 1 College of Landscape Architecture and Horticulture Sciences, Southwest Research Center for Engineering Technology of Landscape Architecture (State Forestry and Grassland Administration), Yunnan Engineering Research Center for Functional Flower Resources and Industrialization, Research and Development Center of Landscape Plants and Horticulture Flowers, Southwest Forestry University, Kunming, 650224 Yunnan China; 2 Key Laboratory for Plant Biodiversity and Biogeography of East Asia, Kunming Institute of Botany, Chinese Academy of Sciences, Kunming, 650201 Yunnan China; 3 Flower Research Institute, Yunnan Academy of Agricultural Sciences, Kunming, 650205 Yunnan China

**Keywords:** *Impatiens uliginosa*, Transcriptome, Nectar spur, Spur development

## Abstract

**Background:**

Spur, a structure capable of producing and storing nectar, not only plays a vital role in the pollination process but also promotes the rapid diversification of some plant lineages, which is considered a key innovation in plants. Spur is the focus of many studies, such as evolution and ecological hypothesis, but the current understanding of spur development is limited. High-throughput sequencing of *Impatiens uliginosa* was carried out to study the molecular mechanism of its spur development, which is believed to provide some insights into the spur development of *Impatiens*.

**Results:**

Transcriptomic sequencing and analysis were performed on spurs and limbs of *I. uliginosa* at three developmental stages. A total of 47.83 Gb of clean data were obtained, and 49,716 unigene genes were assembled. After comparison with NR, Swiss-Prot, Pfam, COG, GO and KEGG databases, a total of 27,686 genes were annotated successfully. Through comparative analysis, 19,356 differentially expressed genes were found and enriched into 208 GO terms and 146 KEGG pathways, among which plant hormone signal transduction was the most significantly enriched pathway. One thousand thirty-two transcription factors were identified, which belonged to 33 TF families such as MYB, bHLH and TCP. Twenty candidate genes that may be involved in spur development were screened and verified by qPCR, such as SBP, IAA and ABP.

**Conclusions:**

Transcriptome data of different developmental stages of spurs were obtained, and a series of candidate genes related to spur development were identified. The importance of genes related to cell cycle, cell division, cell elongation and hormones in spur development was clarified. This study provided valuable information and resources for understanding the molecular mechanism of spur development in *Impatiens*.

**Supplementary Information:**

The online version contains supplementary material available at 10.1186/s12870-022-03894-1.

## Introduction

Floral spur, the tubular outgrowth of a plant petal or sepal, widely exists in a variety of taxa, such as *Impatiens* (Balsaminaceae), *Aquilegia* (Ranunculaceae), *Linaria* (Plantaginaceae), etc. As a structure that produces and stores nectar (or disguised as such), spur plays a vital role in plant pollination by providing rewards to attract pollinators. The interactions with spurs lead to pollinator specialization, which promotes reproductive isolation and speciation in certain plant phylogenetic lineages [1, 2]. Therefore, spur is considered a ‘key innovation’ [3, 4]. Early studies on spurs focused on pollination biology. Two hypotheses based on the study of *Angraecum*, ‘coevolutionary race’ and ‘pollinator shift’, both speculated that with the continuous adaptation of pollinators with longer tongues, spurred plants were more specialized in morphology and function, and short-tongued pollinators would be gradually excluded from the pollination system [5, 6]. Another recent study showed a different view. *Impatiens burtonii* seemed to be more generalized than the specialization of previous studies. The complex structure of spurs in *I. burtonii* separated pollinators’ spatial and temporal niches, thus allowing pollination of both short-tongued and long-tongued visitors [7]. The adaptation and evolution of spurred plants of different groups to natural pressure seem diverse.

Studies on *Aquilegia* and *Linaria* have shown two types of spur development patterns. In *Aquilegia*, the spurs go through two stages of development. At stage 1, local cell divisions around the presumptive nectary gave rise to the nectary cup, namely the nascent spur, and then division activity ceased when the spur was only a small fraction of the final length. Then anisotropic cell elongation brought spur to its final morphology at stage 2 [8]. *Centranthus ruber* also had two stages of spur development that were highly similar to *Aquilegia*. The difference was that although the cell division diminished significantly at the end of the first stage in *C. ruber*, it did not stop completely, and there was still a small amount of cell division activity in the later stages of development [9]. In contrast to this anisotropic elongation dominated development model, in *Linaria*, spur length depended primarily on the number of cells from the initial cell division, although slow anisotropic growth remains throughout development [10]. Spur development includes cell division and anisotropic cell elongation [11]. It plays a role in different populations through unique combination patterns, which makes the development and evolution of spur have different mechanisms in different plant systems.

The mutation of *Hirzina* and *Invaginata* loci led to the ectopic expression of *KNOX*, resulting in a spur-like structure on the petals of *Antirrhinum majus*, which does not have a spur [12]. The homologous of *KNOX* was highly expressed in the petals of *Linaria vulgaris* and *Dactylorhiza fuchsia*, and the introduction of exogenous *KNOX* of *A. majus* and *L. vulgaris* into tobacco produced sac-like protrusions [12, 13]. *KNOX* seems to be the key to regulating the formation of spurs in these plants. However, studies on Papaveraceae showed that spur formation is not significantly related to the expression of *KNOX*, suggesting other possibilities for the molecular mechanism of spur development [14]. The hybridization experiment between *Aquilegia ecalcarata* and spurred species showed that there was a single genetic factor regulating the presence/absence of spur in *Aquilegia*, which was later proved to be *POPOVICH*, a transcription factor encoding C2H2 zinc finger protein [15–17]. Downstream of this locus, multiple genes regulated the variation of spur length, such as *ARF6*, *ARF8*, and so on [18]. In addition, *TCP4* gene regulated the normal development of spur by inhibiting the cell proliferation of the distal part, while *KNOX* was not involved in the main regulation process [19].

Extensive research on spur has been carried out in many aspects. It can be seen that there are differences among systems in terms of interaction with pollinators and mechanisms of development. Spur in each group has its unique evolutionary mode and developmental system in the long process of evolution. As a spurred species, there have been some studies on the pollination biology of *Impatiens* [7, 20], but research on spur development is lacking. De novo transcriptome sequencing was performed on the limb and nectar spur of *I. uliginosa* to explore the molecular mechanism of spur development in *Impatiens*. Some candidate genes involved in spur development were identified by detailed analysis. This study may provide some insights into the spur development mechanism of *Impatiens*.

## Results

### Spur development of *I. uliginosa*

**Fig. 1:**
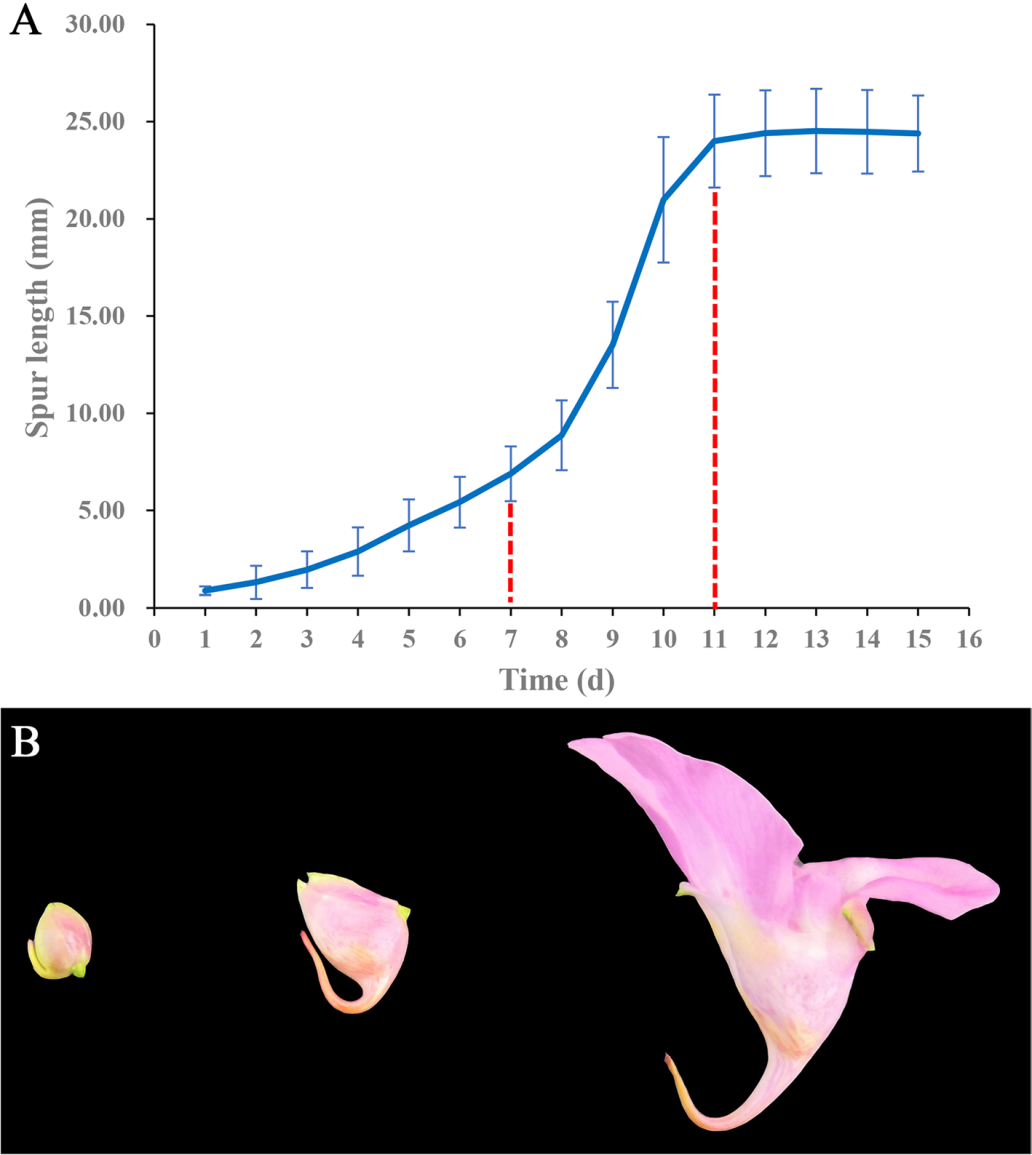
**A** Average growth curve of spur of *I. uliginosa*. The red dashed line indicates the boundary between the three development stages **B** Spur in the early stage, showing a straight appearance (left); Spur in the middle stage, producing an inward curve (middle); Spur at anthesis (right)

The bud with undeveloped spur from each of the 30 well-growing plants of *I. uliginosa* was selected randomly and observed every day. When the bulge of the spur was observed, the day was recorded as day 1. Spur length was measured at the same time every day until the flowers withered. Finally, growth data were obtained for a total of 30 spurs, 4 of which showed great individual differences. The time frame of development of these 4 individuals was significantly shorter than the average level, so the data were not used. The data of the other 26 individuals were used to draw the spur growth curve (Fig. 1A).

According to the growth curve and the morphological characteristics of spur, three development stages were divided. The early stage lasted about 7 days, and spur grew from the bottom of the funnel-shaped labellum to 5 ~ 8 mm long. Different from species with straight spurs, such as *Aquilegia* and *Linaria*, although the spur of *I. uliginosa* also grew in a straight line at this stage, it was oriented upwards. Then, in the middle stage, the growth rate greatly increased, extending to about 25 mm in about 4 days. The spur bent from its junction with the limbs, and the tip of spur was still upward while the part from the connection to the bend grew downward. Some spurs stopped growing completely at the blooming stage, while others continued to elongate by about 1 ~ 2 mm. Spur length basically reached a stable state until the flowers withered (Fig. 1B).

### RNA sequencing and de novo assembly of transcriptome

Transcriptome analysis was performed on spurs and limbs at different developmental stages, which were designated DEC (spur in early stage), DEB (limb in early stage), DMC (spur in middle stage), DMB (limb in middle stage), DAC (spur at anthesis) and DAB (limb at anthesis). A total of 321,843,272 raw reads and 48.6 Gb of raw data were generated. After quality control, 47.83 GB high quality clean data were obtained with approximately 50 million clean reads for each sample. Q30 ranged from 94.95 to 95.83% and GC content was above 43.94%. The mapping ratio of the six samples was more than 81.50% (Table 1).

**Table 1:** Summary of sequencing data of *I. uliginosa* transcriptome

Attributes	DEC	DEB	DMC	DMB	DAC	DAB
Raw reads	50,368,450	56,275,322	53,164,256	50,216,112	53,406,892	58,412,240
Clean reads	50,026,772	55,913,178	52,731,754	49,831,786	52,961,214	58,019,782
Clean bases	7,492,666,959	8,378,452,375	7,899,088,388	7,457,133,163	7,916,885,573	8,688,078,396
Q30(%)	95.83	95.36	95.04	94.95	94.95	95.45
GC content(%)	44.06	44.38	44.15	43.94	44.69	44.21
Total mapped	41,733,944 (83.42%)	47,119,790 (84.27%)	44,451,460 (84.30%)	41,554,626 (83.39%)	43,163,028 (81.50%)	48,458,136 (83.52%)

**Fig. 2:**
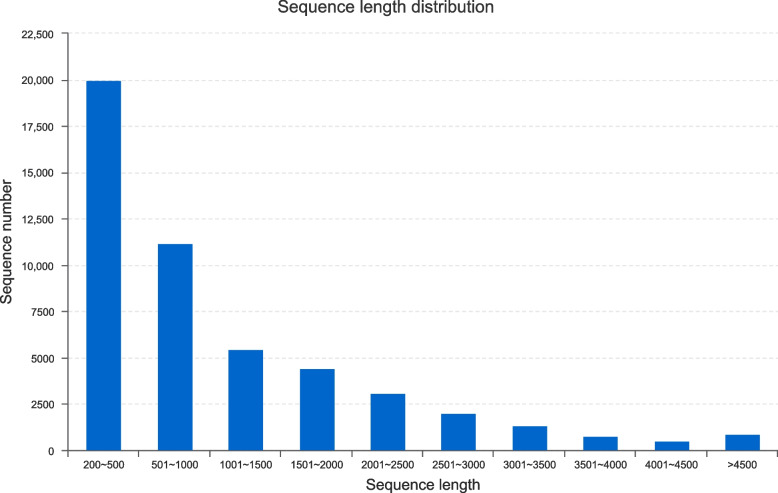
Length distribution of unigenes

De novo assembly was performed on high quality reads, and 86,413 transcripts (119.7 Mb of sequence) were produced after optimization and filtration. The maximal and minimal transcript length was 13,646 and 201 bp respectively, with an average length of 1384.75 bp and N50 value of 2068 bp. A total of 49,716 unigenes (55.9 Mb of sequence) were obtained, the maximum length and minimum length were consistent with the transcripts, with an average length of 1124.51 bp and N50 value of 1928 bp (Table 2). 82% of the unigenes ranged from 200 to 2000 bp, 15% ranged from 2000 to 4000 bp, and 1421 unigenes were over 4000 bp, accounting for 3% of the total (Fig. 2).

**Table 2:** Statistics of transcriptome assembly

Attributes	unigenes	transcripts
Total number	49,716	86,413
Total base	55,906,377	119,660,062
Largest length (bp)	13,646	13,646
Smallest length (bp)	201	201
Average length (bp)	1124.51	1384.75
N50 length (bp)	1928	2068

### Functional annotation

BLAST search (E-value < 1e-5) was used to compare the assembled sequences with NR (NCBI Non-Redundant Protein Sequence Database), Swiss-Prot, Pfam (Protein families), COG (Clusters of Orthologous Groups of proteins), GO (Gene Ontology) and KEGG (Kyoto Encyclopedia of Genes and Genomes) databases to obtain the annotation information of transcriptome. Of all the 49,716 unigenes, 55.69% (27,686 unigenes) were successfully annotated in the six databases (Table 3, Table S1). 25,813 (51.92%) unigenes were aligned to the NR database, 44.54% (11,496 unigenes) of the mapped sequences showed a similarity of more than 80%, and 19,507 unigenes (75.57%) showed high homology (<1E-30). *Camellia sinensis* (6182, 23.95%), *Actinidia chinensis* (4594, 17.8%), *Vitis vinifera* (1189, 4.61%), *Quercus suber* (1039, 4.03%) and *Oryza sativa* (364, 1.41%) were the top five species that showed similarity with unigenes of *I. uliginosa* (Fig. S1). 22,028 and 22,214 unigenes were assigned to Swiss-Prot and Pfam databases respectively.

**Table 3:** Functional annotation of *I. uliginosa* unigenes

Database	Number of unigenes	Percentage
NR	25,813	51.92%
Swiss-Prot	22,028	44.31%
Pfam	22,214	44.68%
COG	25,018	50.32%
GO	21,955	44.16%
KEGG	13,416	26.99%
Total	27,686	55.69%

**Fig. 3:**
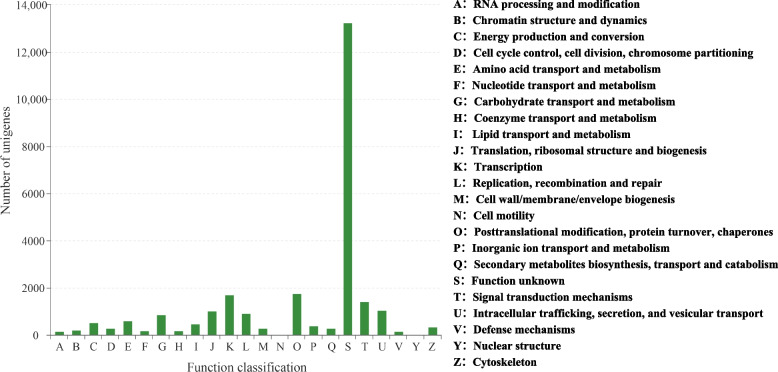
COG classification of unigenes in *I. uliginosa*

**Fig. 4:**
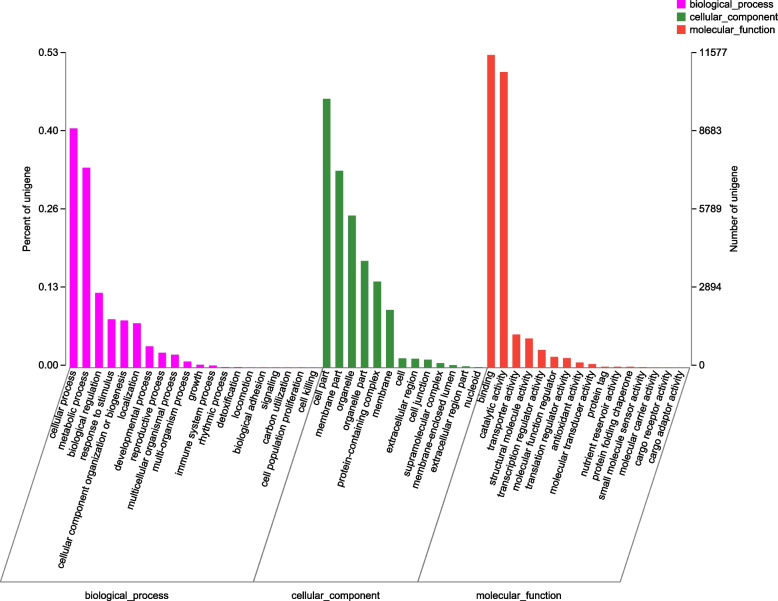
Main GO categories of unigenes in *I. uliginosa* transcriptome

**Fig. 5:**
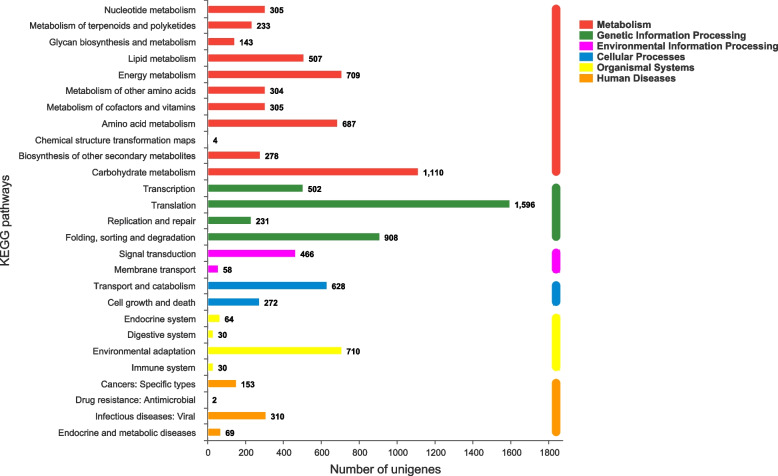
KEGG metabolic pathway of unigenes in *I. uliginosa*

A total of 25,018 unigenes were assigned to the 23 COG categories, 13,235 of which had poor characteristics and were matched to unknown functions. Among the effectively annotated unigenes, the largest group was ‘posttranslational modification, protein turnover, chaperones’, followed by ‘transcription’ and ‘signal transduction mechanisms’ (Fig. 3). Twenty-one thousand nine hundred fifty-five unigenes were classified into at least one GO term. ‘Cellular process’ and ‘metabolic process’ were the most abundant subcategories for biological process (BP). Within the cellular component category (CC), most unigenes were clustered in ‘cell part’. At the level of molecular function (MF), ‘binding’ and ‘catalytic activity’ were the most significant subcategories (Fig. 4). Thirteen thousand four hundred sixteen unigenes successfully annotated to KEGG were classified into 27 pathways in five main categories. Most genes were associated with the categories ‘metabolism’ and ‘genetic information processing’, and pathways such as ‘translation’, ‘carbohydrate metabolism’ and ‘folding, sorting and degradation’ were the most representative (Fig. 5).

### Differential gene expression

**Fig. 6:**
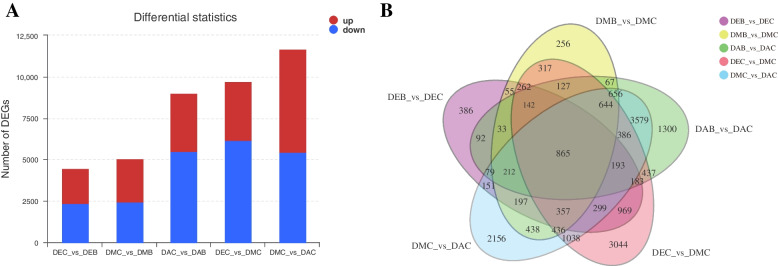
**A** Differentially expressed genes in different developmental stages and tissues **B** Venn diagram of DEGs

In order to explore the genes that may be involved in regulating the spur development of *I. uliginosa*, the differential expression between different developmental stages and tissues was analyzed. The results showed that a total of 19,356 genes were differentially expressed among five groups (p-adjust < 0.001, |log2FC| ≥1) (Fig. 6A). During the three developmental stages, 4475, 5064 and 8995 genes were differentially expressed between limbs and spurs, with 2347, 2440, 5486 up-regulated and 2128, 2624, 3509 down-regulated, respectively. In spurs, there were 9699 DEGs (differential expression genes) between the early stage and the middle stage, with 3533 up-regulated and 6166 down-regulated, and 11,686 DEGs between the middle stage and anthesis, with 6237 up-regulated and 5449 down-regulated. There were 865 DEGs shared among these five groups (Fig. 6B).

### Enrichment analysis of DEGs

**Fig. 7:**
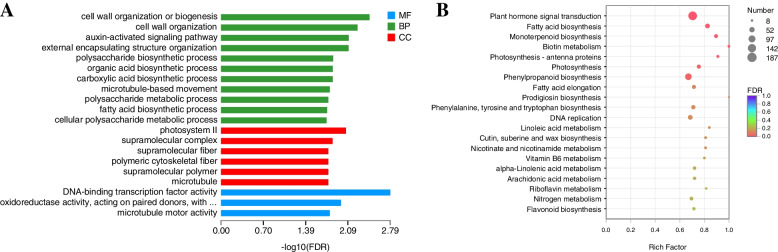
**A** The top 20 enriched GO terms of DEGs **B** The top 20 enriched KEGG pathways of DEGs

Functional enrichment analysis of DEGs was carried out to understand their possible roles in spur development. The results indicated that all 19,356 DEGs were matched to 208 GO terms, of which 30 terms were significantly enriched (p-adjust < 0.05) (Table S2). ‘cell wall organization or biogenesis’, ‘photosystem II’ and ‘DNA-binding transcription factor activity’ were the most significantly enriched terms, respectively (Fig. 7A). Notably, the main enrichment function of DEGs changed at different developmental stages. In the early stage (DEB_vs_DEC), ‘photosynthesis, light harvesting’, ‘regulation of cell cycle’, ‘protein-chromophore linkage’ and ‘auxin-activated signaling pathway’ were very significant biological processes, while in the middle stage (DMB_vs_DMC), ‘metal ion transport’ was one of the most abundant terms. ‘External encapsulating structure organization’ and ‘cell wall organization’ were very prominent in both the middle and flowering stages (DMB_vs_DMC, DAB_vs_DAC, DMC_vs_DAC), while ‘hormone-mediated signaling pathway’ played an important role in the whole development process (Fig. S2).

KEGG enrichment analysis of DEGs indicated that all 19,356 unigenes were assigned to 146 KEGG pathways, and 7 of them showed significant enrichment (p-adjust < 0.05) (Fig. 7B, Table S3). Further analysis of DEGs in different stages and tissues showed that ‘Plant hormone signal transduction’ was the most abundant pathway in all stages of spur development, while ‘Photosynthesis - antenna proteins’, ‘Phenylpropanoid biosynthesis’, ‘Monoterpenoid biosynthesis’, ‘Plant-pathogen interaction’, ‘Photosynthesis’ and ‘Fatty acid elongation’ were very prominent in both early and middle stages (DEB_vs_DEC, DMB_vs_DMC, DEC_vs_DMC). ‘Homologous recombination’ and ‘MAPK signaling pathway’ showed significant enrichment only in the early stage (DEB_vs_DEC), while ‘Steroid biosynthesis’ and ‘Cutin, suberine and wax biosynthesis’ showed significant enrichment only in the middle stage (DMB_vs_DMC). The pathways significantly enriched at the flowering stage (DAB_vs_DAC) were basically different from other groups, such as ‘Thermogenesis’, ‘Fatty acid degradation’ and ‘Spliceosome’, etc. (Fig. S3).

### Identification of transcription factors

**Fig. 8:**
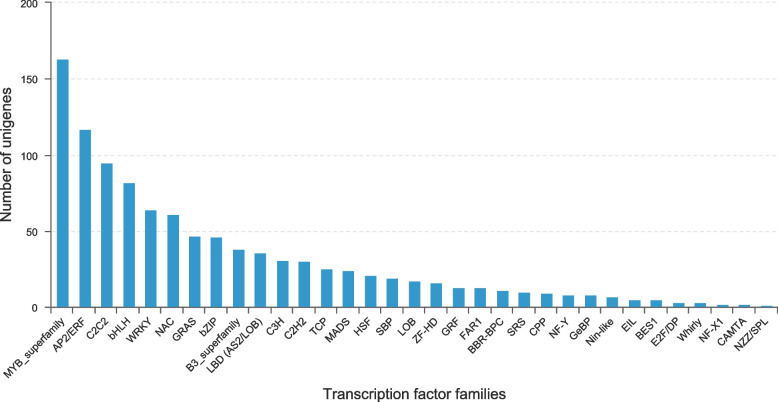
Families of transcription factors identified in the *I. uliginosa* transcriptome

**Fig. 9:**
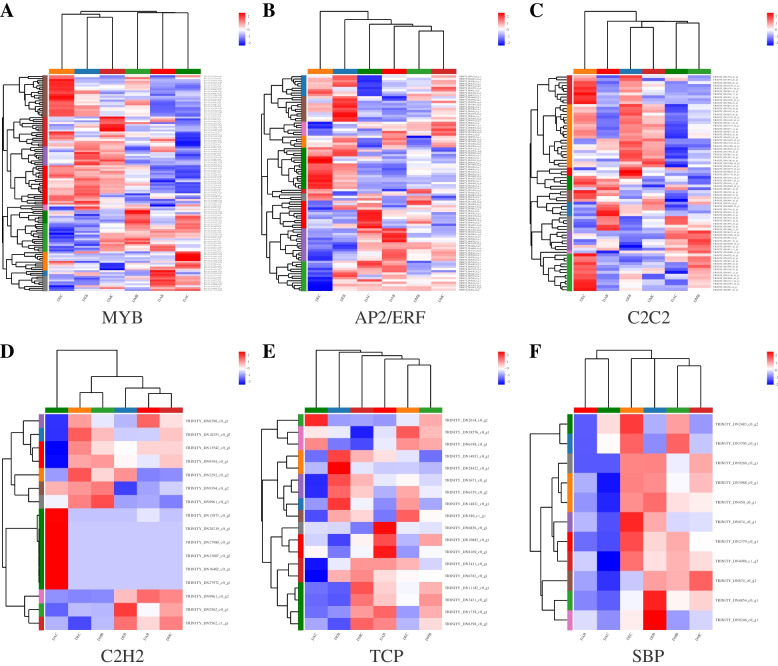
Cluster heat maps of DEGs in transcription factor families **A** MYB **B** AP2/ERF **C** C2C2 **D** C2H2 **E** TCP **F** SBP

Transcription factors were predicted by analyzing the domain information in the transcripts. A total of 1032 unigenes were annotated as transcription factors, belonging to 33 transcription factor families (Fig. 8). The most prominent family is MYB, which is assigned 163 genes, followed by AP2/ERF (117), C2C2 (95), bHLH (82) and WRKY (64). In addition, C2H2, TCP and GRF families have been proved to play an important role in spur development in *Aquilegia*. SBP family was well represented in the transcriptome of *I. uliginosa*. Of all the transcription factors, 726 genes were differentially expressed across tissues, the most significant being up to 2280-fold (Table S4). Most genes of MYB and C2C2 had high expression in the early stage, gradually down-regulated in the middle stage and blooming stage (Fig. 9A, C). AP2/ERF TFs showed two trends, one with higher expression in the early stage, down-regulated in the middle stage and blooming stage, the other with lower expression in the early and middle stages, then up-regulated in the flowering stage (Fig. 9B). Some TFs of C2H2 were highly expressed only in spur at anthesis but very low within other tissues, while the other TFs showed significant differences in spur and limb at each stage (Fig. 9D). The expression levels of TCP and SBP TFs were relatively high in the early and middle stages, but low in anthesis (Fig. 9E, F).

### Candidate genes involved in petal development

**Fig. 10:**
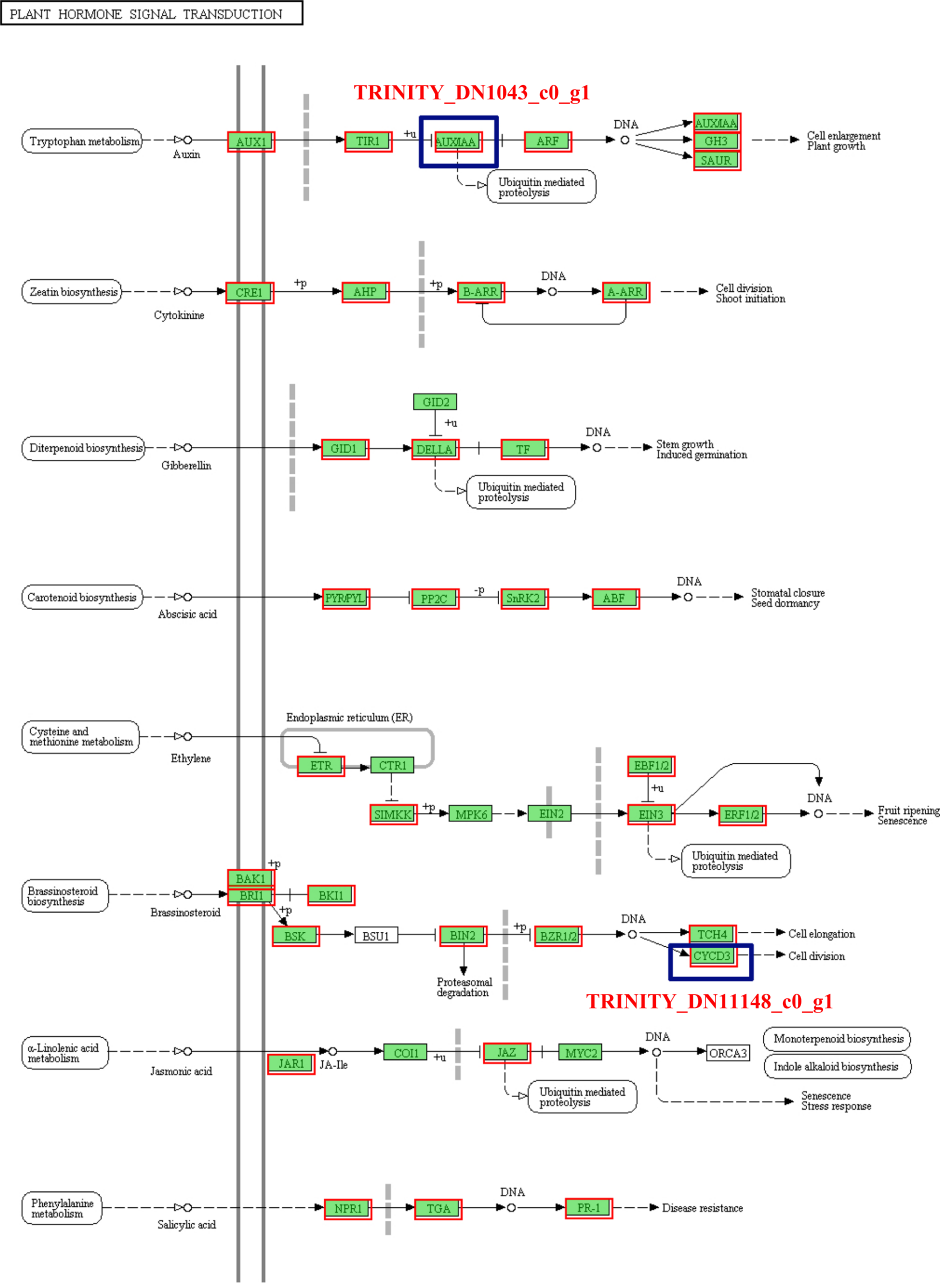
The pathway of plant hormone signal transduction, two thick blue boxes represent the location of candidate genes

Twenty candidate genes involved in spur development were identified according to their function and differential expression in different tissues (Table S5). These included two genes annotated as Extensin, with extremely high expression levels (TPM > 2000) in the early spur and significant differences from the limb (fold change > 220); three SBP transcription factors that proved to play a regulatory role in flower development; one Cyclin gene (TRINITY_DN11148_c0_g1) and one IAA gene (TRINITY_DN1043_c0_g1) located in ‘plant hormone signal transduction’, the most significantly enriched pathway (Fig. 10) [21]. Some genes that have been confirmed to regulate the development of spur in species such as *Aquilegia* and *Linaria* have also performed well in *I. uliginosa*, including three Aquaporin genes, one *KNOX* gene and two *TCP4* genes. In addition, there are three genes encoding Dof zinc finger protein, one cell division cycle gene, one ABP gene, one floral-binding protein gene and one Expansin gene.

### qRT-PCR validation of the candidate genes

**Fig. 11:**
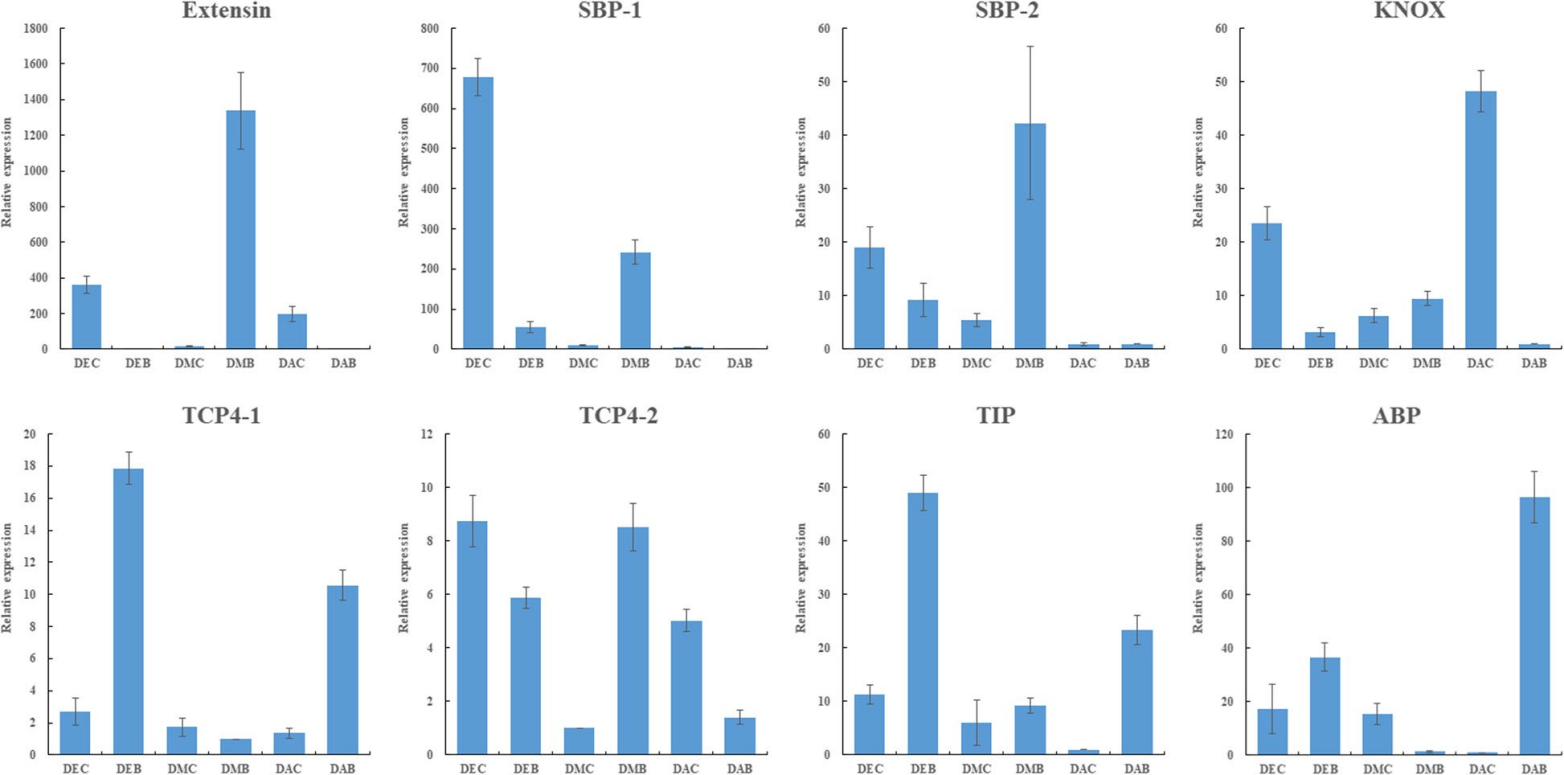
Expression analysis of eight candidate genes in 6 tissues by qRT-PCR. DEC: spur in the early stage, DEB: limb in the early stage, DMC: spur in the middle stage, DMB: limb in the middle stage, DAC: spur at anthesis, DAB: limb at anthesis

Eight candidate genes were selected for qRT-PCR to verify the accuracy of transcriptome data (Fig. 11, Table S6). The qPCR results showed that Extensin, KNOX and TCP4–2 [22] represented similar expression trends, with expression differences between spurs and limbs in all three stages. The expression of spurs was higher than that of limbs in the early and anthesis stages, while in the middle stage, it was the limb higher than the spur. The expression trends of SBPs in the early and middle stages were similar to Extensin but were very low in both the spur and limb at the flowering stage. The expression levels of TCP4–1 [22], TIP [23] and ABP [24] genes in limbs were significantly higher than spurs in the early stages but did not show much difference in the middle stage. All the verified genes had similar expression patterns to the transcriptome data, and there was a strong correlation between their expression levels (Fig. S4). Therefore, the transcriptome data can be used to analyze genes related to spur development.

## Discussion

Spur plays a vital role in the pollination process. Its special structure will not only affect the preferences and behavior of pollinators but also further affect the composition of the pollination system, and it may play a role in ecological control [25–28]. The morphology of spur itself may also evolve and change due to the interaction with pollinators. This trait has rapidly diversified the species of some plant lineages, making it known as a ‘key innovation’. These characteristics made spur became the focus of many botanists’ research. All species in *Impatiens* contain the spur structure and have abundant morphological differences between species, which is an ideal population for studying spur. However, the current research on the spur of *Impatiens* mainly focused on pollination biology and morphological structure [7, 20, 29], and there was no relevant report on the mechanism of spur development. According to previous studies, it can be seen that there was no unified pattern of spur development among different plant groups, such as *Aquilegia* and *Linaria*. *Impatiens* has different curved spur from *Aquilegia*, *Linaria* and *Centranthus ruber*, which makes it more likely to have a distinctive development model. Therefore, *I. uliginosa* was selected for transcriptome sequencing analysis to dig out the key genes related to spur development to conduct an in-depth study on the mechanism of spur development of *Impatiens*.

Studies on the spur of *Aquilegia* have confirmed that genes with differences between spur and blade tissue are likely to be involved in the regulation of spur development [17–19]. The labellum of *I. uliginosa* was similar to the petals of *Aquilegia* to some extent in structure, that is, a tubular spur was extended at the bottom of the funnel-shaped petals. Therefore, referring to the research method of *Aquilegia*, spur and limb tissues of different development stages were taken for transcriptome sequencing analysis. After quality control and assembly evaluation, a total of 47.83 GB of high-quality cleaning data and 49,716 unigenes were obtained, with an average length of 1124.51 bp and N50 of 1928 bp. These length attributes of unigenes had better performance than the published data of *Camellia Sasanqua* [30], Rhododendron Rex [31], *Ocimum Americanum* [32] and Gmelina area [33], and the good quality of this transcriptome data was available for the subsequent analysis.

The expression of genes in tissues at different developmental stages and positions (DEB vs DEC, DMB vs DMC, DAB vs DAC, DEC vs DMC, DMC vs DAC) were analyzed. Among all the 49,716 unigenes, a total of 19,356 genes showed expression differences, of which 865 genes showed differences in all five groups. GO and KEGG enrichment analyses were carried out to further explore how these DEGs play a role in spur development. The results showed that in the early stage, DEGs were significantly enriched in terms such as ‘regulation of cell cycle’, ‘auxin-activated signaling pathway’, ‘zeatin biosynthesis’, ‘DNA replication’ and ‘brassinosteroid biosynthesis’, which proved that cell division and growth activities were vigorous in the early stage, which was consistent with studies of *Aquilegia* and *Linaria* on spur [10, 19, 34]. Moreover, many terms related to photosynthesis were significantly enriched in the early stage. In the middle stage, the number of terms and pathways related to cell division and growth activities decreased, with significantly reduced enrichment. The number of terms and genes related to photosynthesis also decreased, while the biological activities related to ‘cell wall organization’ were significantly enriched. During anthesis, the terms related to photosynthesis disappeared, while the biological activities related to ‘cell wall’ increased. ‘hormone-mediated signaling pathway’ and ‘plant hormone signal transduction’ were consistently the most significantly enriched terms across the three developmental stages.

Transcription factors play an important role in regulating plant growth and development and stress resistance. MYB TFs regulated cell cycle, early inflorescence development and seed germination [35–37], and also participated in the synthesis of metabolites such as flavonol and anthocyanin in plants [38, 39]. AP2/ERF played a key regulatory role in floral development, such as promoting the establishment of floral meristem, regulating the development of sepals and petals, and regulating the expression of genes related to flower development [40–43]. SBP TFs participated in regulating the development of floral organs such as megaspore, microspore, pollen tube and stamen filament, and it also affected the morphology of inflorescences and leaves [44–46]. C2H2 gene controlled the presence or absence of spur in *Aquilegia* [17], and TCP gene controlled the proper directed growth of spur cells [19], two known transcription factors that can affect spur growth.

In this study, a total of 1032 transcription factors belonging to 33 families were identified, of which 726 genes in 32 families were differentially expressed. DEGs in these TFS were likely to impact the regulation of spur development. MYB and AP2/ERF were the two TF families with the most abundant genes. MYB DEGs were most highly expressed in the early stage and gradually declined in the middle and flowering stages, suggesting that they might be involved in the early development of spur and cell cycle regulation. AP2/ERF genes showed two expression trends: one was highly expressed at the early stage and down-regulated at the middle and anthesis stage; the other was low expressed at the early and middle stages and up-regulated at the anthesis stage, suggesting that AP2/ERF might play an important role in the differentiation of spur. SBP DEGs have high expression in the early stage, down-regulated in the middle stage, and very low expression in the flowering stage. There was a very significant difference between spur and limb, and it was speculated that it would affect the morphogenesis of spur. C2H2 and TCP families also showed significant differences and high expression among tissues.

Twenty candidate genes that may be related to spur development were screened, and qRT-PCR was performed on eight of them to verify the reliability of transcriptome data. The results showed that these genes were significantly differentially expressed at different developmental stages and sites. Extension is the most abundant structural protein in the primary cell wall of dicotyledons, which can increase the cell wall’s strength and stiffness, control the cell wall’s elongation and regulate plant morphogenesis [47, 48]. Extension may be involved in the anisotropic elongation of spur cells. Aquaporins could efficiently transport water molecules and mediate the transport of other small molecular substances, nutrient elements and metal ions [49, 50]. The water potential gradient generated by the accumulation of solutes regulates the turgor of cells, thus promoting cell division and growth. Aquaporin TIPs and PIPs have been proved to be positively correlated with cell expansion [51, 52]. Dof genes were not only transcription regulators of cell cycle genes but also participated in the auxin response by combining with other genes [53, 54]. The development of spur is mainly a process of cell division and anisotropic elongation [11]. All of these genes involved in cell elongation, cell division, cell cycle and hormone regulation and response affected cell division and elongation activities directly or indirectly. They most likely play an essential role in the formation and development of spur.

## Conclusions

This study performed transcriptome sequencing on the spur and limb tissue at various development stages of *I. uliginosa*. Through clustering and functional enrichment analysis of 19,356 differentially expressed genes, candidate genes related to spur development were screened. These data revealed that hormones play an important role in spur development, and genes regulating cell elongation, cell division and cycle are the most critical factors affecting spur development. This is the first study on the molecular mechanism of spur development in *Impatiens*, providing necessary information and a theoretical basis for the mechanism of spur development in dicotyledons.

## Methods

### Plant materials

Seeds of the wild population of *I. uliginosa* were collected from Laoyuhe Wetland Park in Kunming and cultivated into plants in the greenhouse of Southwest Forestry University. The growth conditions were maintained at 18 ~ 25 °C and 11 ~ 13 hours of daylight.

### Observation of spur growth

One flower was randomly selected from each plant for a total of 30 bioreplicates. The length of each spur was measured at 9:00 a.m. every day with three technical replicates. Measurements were performed continuously for 10 to 15 days, depending on the developmental duration of the individual. Growth curves were plotted according to the observed data. Three developmental stages were determined by observing the growth curve of the spur of *I. uliginosa*.

### RNA sequencing and de novo assembly of transcriptome

Referring to Yant et al. [19], tissues from spur and limb of three developmental stages were collected and removed tissues from the connection section (1 ~ 2 mm, Fig. S5). The dissected tissues were stored in liquid nitrogen immediately, and each sample was mixed with more than three biological replicates. Total RNA was extracted using Plant RNA Purification Reagent for plant tissue (Invitrogen, Carlsbad, CA, USA) and genomic DNA was removed using DNase I (Takara). The RNA-seq transcriptome libraries were prepared using the TruSeq™ RNA sample preparation Kit (Illumina, San Diego, CA). After being quantified by TBS380 (Picogreen), the RNAseq libraries were sequenced in a single lane on an Illumina Hiseq Xten/NovaSeq 6000 sequencer (Illumina, San Diego, CA) for 2 × 150 bp paired-end reads.

The raw paired-end reads were trimmed and quality controlled by SeqPrep (https://github.com/jstjohn/SeqPrep) and Sickle (https://github.com/najoshi/sickle) with default parameters. De novo assembly of the clean data was performed with Trinity (http://trinityrnaseq.sourceforge.net/), then the assembled transcripts were homologously clustered, and the longest transcript in each cluster was designated as unigene [55]. The assembled sequences were optimized and filtered using TransRate (http://hibberdlab.com/transrate/), CD-HIT (http://weizhongli-lab.org/cd-hit/) was used to remove redundant and similar sequences, and BUSCO (Benchmarking Universal Single-Copy Orthologs, http://busco.ezlab.org) was used to evaluate the assembly integrity of the transcriptome.

### Functional annotation

All the assembled transcripts were searched against the NR, Swiss-Prot, Pfam and COG databases to retrieve function annotations. BLASTX was used to identify the proteins that had the highest sequence similarity with the given transcripts. A typical cut-off E-value was set to less than 1.0 × 10^− 5^. BLAST2GO (http://www.blast2go.com/b2ghome) [56] program was used to get GO annotations of unique assembled transcripts for describing biological processes, molecular functions and cellular components. Metabolic pathway analysis was performed using the KEGG database (http://www.genome.jp/kegg/) [21].

### Differential expression analysis and functional enrichment

The expression level of gene and transcript was calculated using the TPM (transcripts per million reads) / FPKM (fragments per kilobases per million reads) method, and the abundances of gene and transcript were quantified using RSEM (http://deweylab.biostat.wisc.edu/rsem/) [57]. Differential expression analysis was performed using the DESeq2 [58]/DEGseq [59]/EdgeR [60] with Q value ≤0.05, genes with |log2FC| > 1 and Q value <= 0.05(DESeq2 or EdgeR) /Q value <= 0.001(DEGseq) were considered to be significantly different expressed.

GO and KEGG functional-enrichment analysis was performed on DEGs. Goatools (https://github.com/tanghaibao/GOatools) was used for GO functional enrichment analysis and KOBAS (http://kobas.cbi.pku.edu.cn/home.do) was used for KEGG pathway analysis [61]. Fisher exact test was used for calculation, and *P* values were corrected by Bonferroni and BH (FDR) method. The threshold of the corrected *P*-value was 0.05.

### Identification of transcription factors and cluster analysis

By HMMER analysis, the domain information of transcripts was compared with the database PlantTFDB (http://planttfdb.cbi.pku.edu.cn/) to obtain the homologous transcription factor information for gene transcription factor prediction and family analysis. Clustering analysis of differentially expressed transcription factors was performed using hierarchical clustering methods.

### qRT-PCR analysis of gene expression

Total RNA was extracted from the spur and limb at three developmental stages using E.Z.N.A.® Plant RNA Kit (Omega). The first-strand cDNAs were synthesized using EasyScript® One-Step gDNA Removal and cDNA Synthesis SuperMix (TransGen) and used as templates. qRT-PCR was performed on a LightCycler® 480 II Real-Time Quantitative PCR Detection System (Roche) using Hieff® qPCR SYBR® Green Master Mix (Yeasen). The amplification primers for qRT-PCR were shown in supplementary data Table S7, and *IuActin* was used as a reference gene. Fluorescence quantitative PCR detection was carried out by a three-step method. Each sample had three repetitions. The amplification procedure was as follows: initial denaturation at 95 °C for 5 min, 40 cycles of denaturation at 95 °C for 10 s, annealing at 60 °C for 20 s, extension at 72 °C for 20 s. The comparative cycle threshold (ct) method was used to calculates gene expression levels.

## Supplementary Information

**Additional file 1.**

**Additional file 2.**

**Additional file 3.**

**Additional file 4.**

**Additional file 5.**

**Additional file 6.**

**Additional file 7.**

**Additional file 8.**

**Additional file 9.**

**Additional file 10.**

**Additional file 11.**

**Additional file 12.**

## Data Availability

All data generated in this article are included within the article and its additional files.

## Abbreviations

NR: NCBI Non-Redundant Protein Sequence Database
Pfam: Protein Families
COG: Clusters of Orthologous Groups of proteins
GO: Gene Ontology
KEGG: Kyoto Encyclopedia of Genes and Genomes
DEGs: Differential Expression Genes
