# Supplementary material for: De novo transcriptome sequencing of Impatiens uliginosa and the analysis of candidate genes related to spur development
Source: BMC Plant Biol. 2022 Dec 1;22:553. doi: 10.1186/s12870-022-03894-1 (PMC9713998; doi:10.1186/s12870-022-03894-1)
Supplement: Supplementary file 1 — Additional file 1. [file 12870_2022_3894_MOESM1_ESM.pdf]

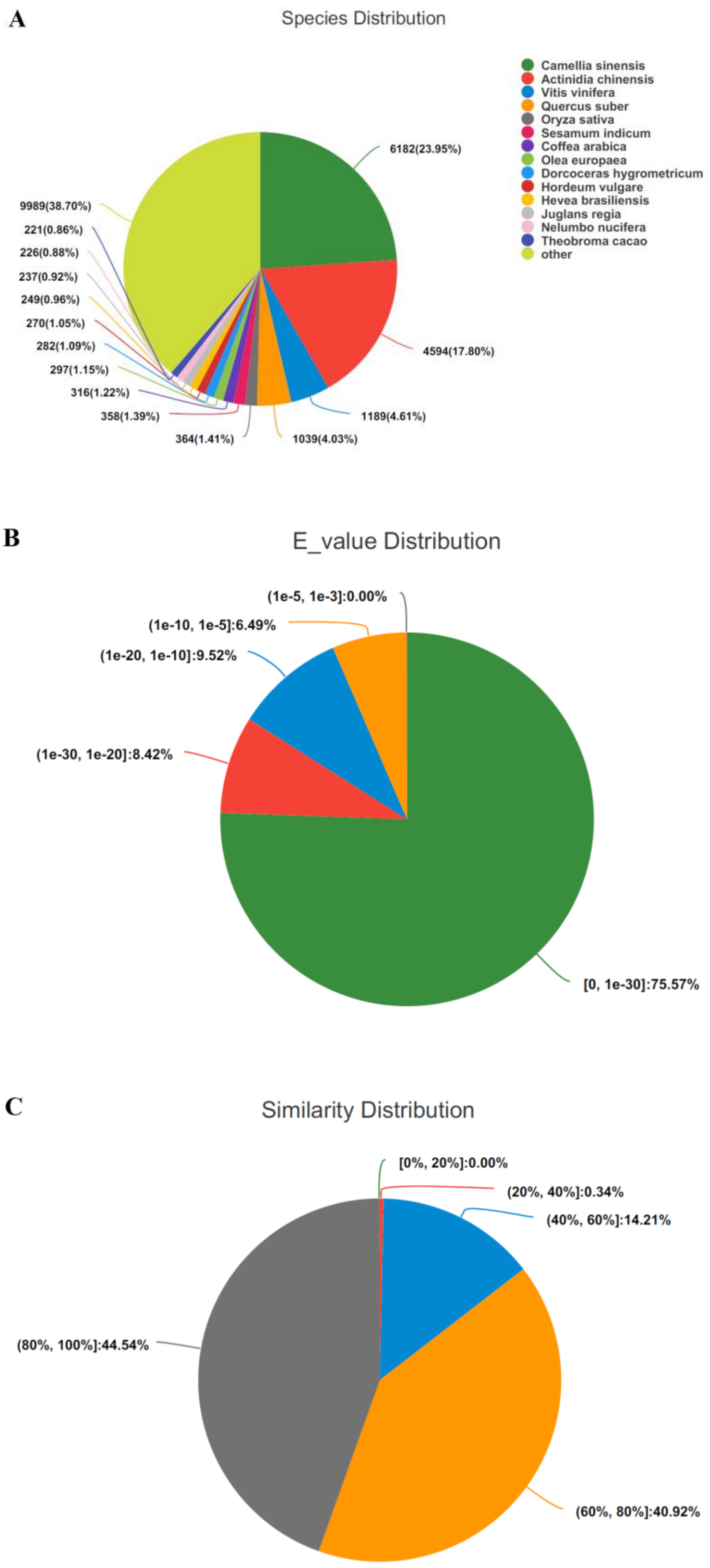

Figure S1 NR annotation information (A) Species distribution map (B) e-value distribution map (C) similarity distributions.
