# Supplementary material for: De novo transcriptome sequencing of Impatiens uliginosa and the analysis of candidate genes related to spur development
Source: BMC Plant Biol. 2022 Dec 1;22:553. doi: 10.1186/s12870-022-03894-1 (PMC9713998; doi:10.1186/s12870-022-03894-1)
Supplement: Supplementary file 2 — Additional file 2. [file 12870_2022_3894_MOESM2_ESM.pdf]

A

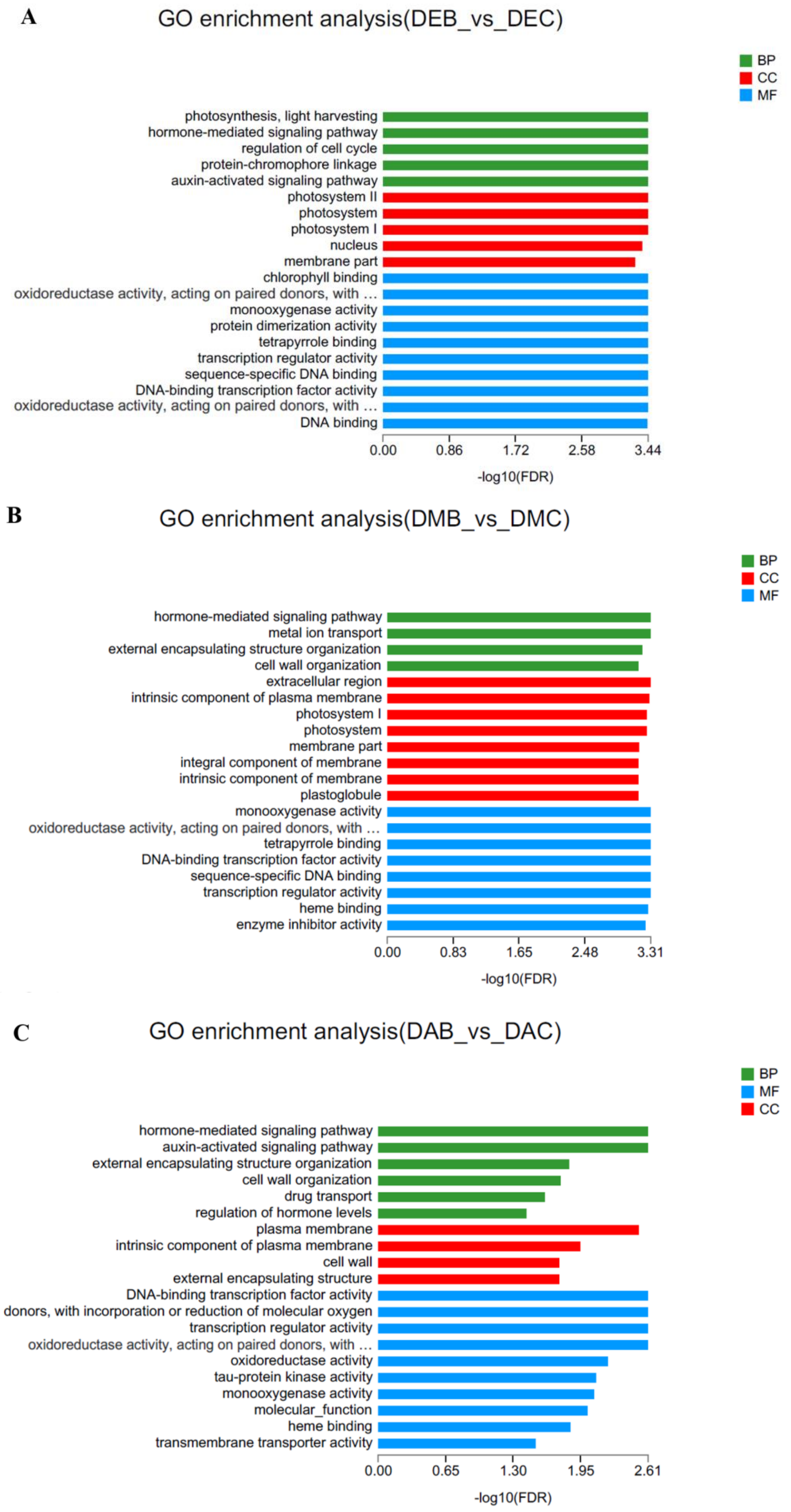
GO enrichment analysis(DEB_vs_DEC)
BP
CC
MF
photosynthesis, light harvesting
hormone-mediated signaling pathway
regulation of cell cycle
protein-chromophore linkage
auxin-activated signaling pathway
photosystem II
photosystem
photosystem I
nucleus
membrane part
chlorophyll binding
oxidoreductase activity, acting on paired donors, with ...
monooxygenase activity
protein dimerization activity
tetrapyrrole binding
transcription regulator activity
sequence-specific DNA binding
DNA-binding transcription factor activity
oxidoreductase activity, acting on paired donors, with ...
DNA binding
0.00
0.86
1.72
2.58
3.44
-log10(FDR)
GO enrichment analysis(DMB_vs_DMC)
BP
CC
MF
hormone-mediated signaling pathway
metal ion transport
external encapsulating structure organization
cell wall organization
extracellular region
intrinsic component of plasma membrane
photosystem I
photosystem
membrane part
integral component of membrane
intrinsic component of membrane
plastoglobule
monooxygenase activity
oxidoreductase activity, acting on paired donors, with ...
tetrapyrrole binding
DNA-binding transcription factor activity
sequence-specific DNA binding
transcription regulator activity
heme binding
enzyme inhibitor activity
0.00
0.83
1.65
2.48
3.31
-log10(FDR)
GO enrichment analysis(DAB_vs_DAC)
BP
MF
CC
hormone-mediated signaling pathway
auxin-activated signaling pathway
external encapsulating structure organization
cell wall organization
drug transport
regulation of hormone levels
plasma membrane
intrinsic component of plasma membrane
cell wall
external encapsulating structure
DNA-binding transcription factor activity
donors, with incorporation or reduction of molecular oxygen
transcription regulator activity
oxidoreductase activity, acting on paired donors, with ...
oxidoreductase activity
tau-protein kinase activity
monooxygenase activity
molecular_function
heme binding
transmembrane transporter activity
0.00
0.65
1.30
1.95
2.61
-log10(FDR)

B

C

D

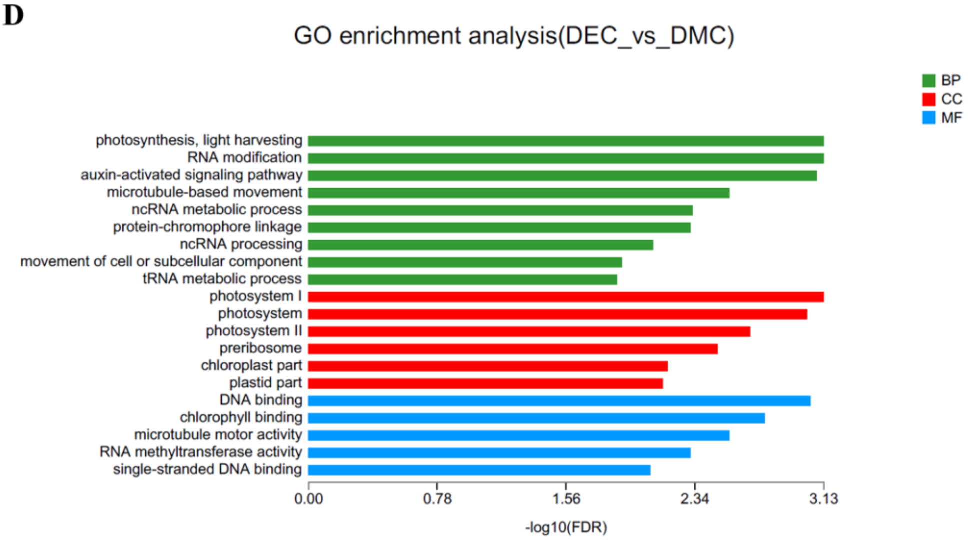

E

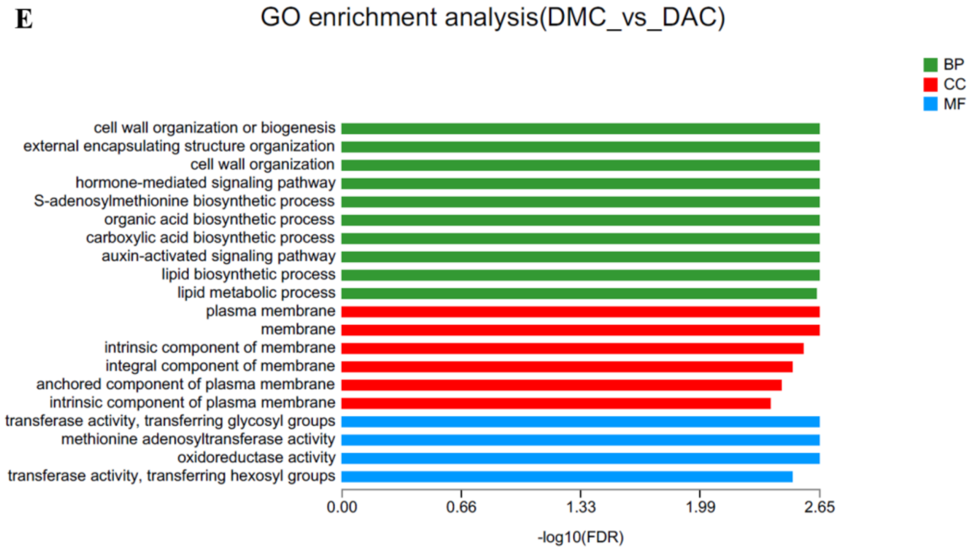

Figure S2 The top 20 enriched GO terms of DEGs in different groups, the ordinate represents GO terms, -log10(FDR) represents the significance level of enrichment. (A) GO enrichment analysis of DEGs between DEB and DEC (B) GO enrichment analysis of DEGs between DMB and DMC (C) GO enrichment analysis of DEGs between DAB and DAC (D) GO enrichment analysis of DEGs between DEC and DMC (E) GO enrichment analysis of DEGs between DMC and DAC.
