# Supplementary material for: De novo transcriptome sequencing of Impatiens uliginosa and the analysis of candidate genes related to spur development
Source: BMC Plant Biol. 2022 Dec 1;22:553. doi: 10.1186/s12870-022-03894-1 (PMC9713998; doi:10.1186/s12870-022-03894-1)
Supplement: Supplementary file 3 — Additional file 3. [file 12870_2022_3894_MOESM3_ESM.pdf]

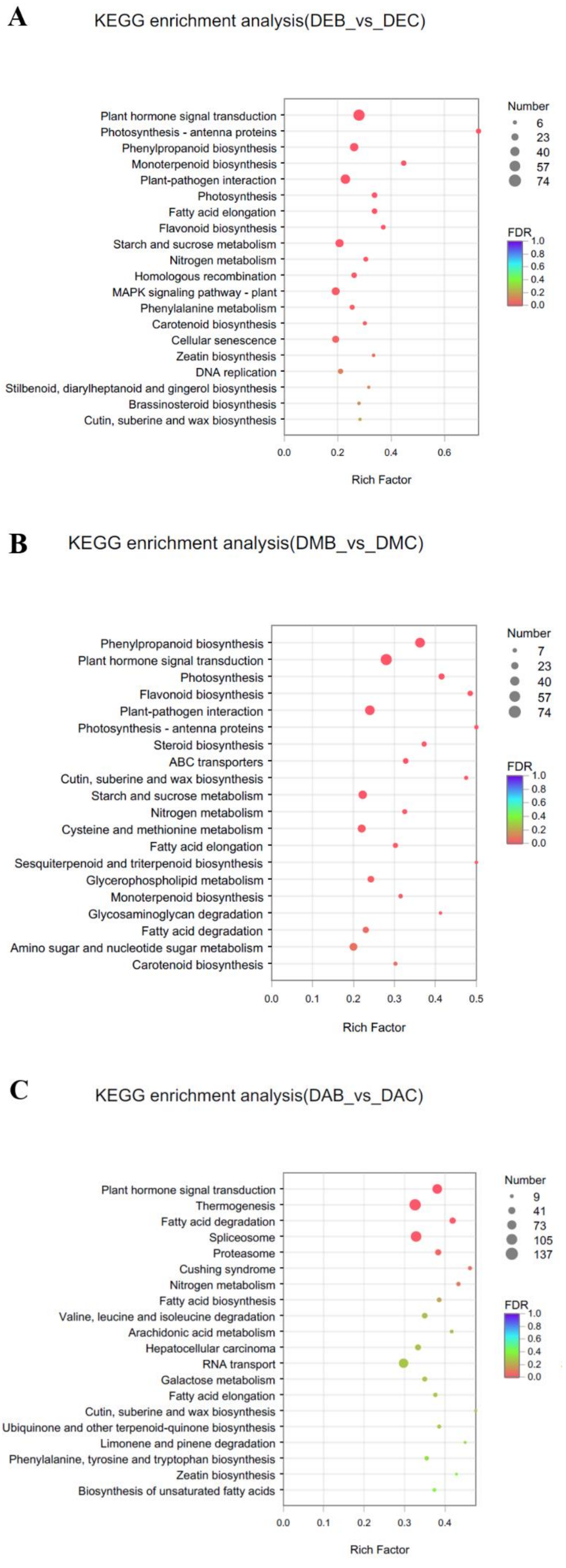
A
KEGG enrichment analysis(DEB_vs_DEC)
Plant hormone signal transduction
Photosynthesis - antenna proteins
Phenylpropanoid biosynthesis
Monoterpenoid biosynthesis
Plant-pathogen interaction
Photosynthesis
Fatty acid elongation
Flavonoid biosynthesis
Starch and sucrose metabolism
Nitrogen metabolism
Homologous recombination
MAPK signaling pathway - plant
Phenylalanine metabolism
Carotenoid biosynthesis
Cellular senescence
Zeatin biosynthesis
DNA replication
Stilbenoid, diarylheptanoid and gingerol biosynthesis
Brassinosteroid biosynthesis
Cutin, suberine and wax biosynthesis
0.0 0.2 0.4 0.6
Rich Factor
Number
6
23
40
57
74
FDR
1.0
0.8
0.6
0.4
0.2
0.0
B
KEGG enrichment analysis(DMB_vs_DMC)
Phenylpropanoid biosynthesis
Plant hormone signal transduction
Photosynthesis
Flavonoid biosynthesis
Plant-pathogen interaction
Photosynthesis - antenna proteins
Steroid biosynthesis
ABC transporters
Cutin, suberine and wax biosynthesis
Starch and sucrose metabolism
Nitrogen metabolism
Cysteine and methionine metabolism
Fatty acid elongation
Sesquiterpenoid and triterpenoid biosynthesis
Glycerophospholipid metabolism
Monoterpenoid biosynthesis
Glycosaminoglycan degradation
Fatty acid degradation
Amino sugar and nucleotide sugar metabolism
Carotenoid biosynthesis
0.0 0.1 0.2 0.3 0.4 0.5
Rich Factor
Number
7
23
40
57
74
FDR
1.0
0.8
0.6
0.4
0.2
0.0
C
KEGG enrichment analysis(DAB_vs_DAC)
Plant hormone signal transduction
Thermogenesis
Fatty acid degradation
Spliceosome
Proteasome
Cushing syndrome
Nitrogen metabolism
Fatty acid biosynthesis
Valine, leucine and isoleucine degradation
Arachidonic acid metabolism
Hepatocellular carcinoma
RNA transport
Galactose metabolism
Fatty acid elongation
Cutin, suberine and wax biosynthesis
Ubiquinone and other terpenoid-quinone biosynthesis
Limonene and pinene degradation
Phenylalanine, tyrosine and tryptophan biosynthesis
Zeatin biosynthesis
Biosynthesis of unsaturated fatty acids
0.0 0.1 0.2 0.3 0.4
Rich Factor
Number
9
41
73
105
137
FDR
1.0
0.8
0.6
0.4
0.2
0.0

D

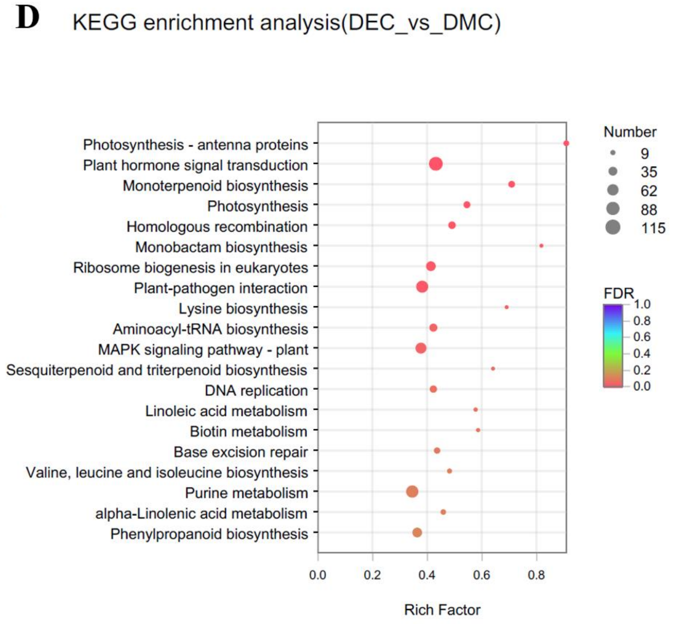

E

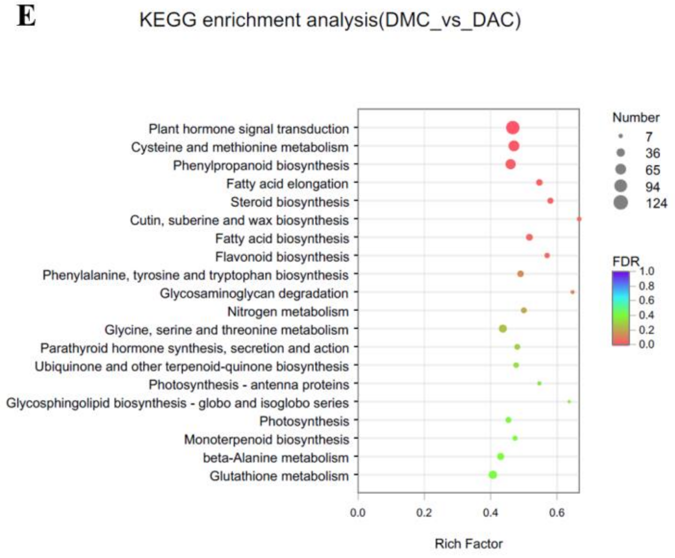

Figure S3 The top 20 enriched KEGG pathways of DEGs in different groups, the ordinate represents KEGG pathways, the rich factor represents the significant level of enrichment, the size of the point represents the number of unigenes, the color of the point corresponds to the range of q-value. (A) KEGG enrichment analysis of DEGs between DEB and DEC (B) KEGG enrichment analysis of DEGs between DMB and DMC (C) KEGG enrichment analysis of DEGs between DAB and DAC (D) KEGG enrichment analysis of DEGs between DEC and DMC (E) KEGG enrichment analysis of DEGs between DMC and DAC.
