# Supplementary figures and images for: De novo transcriptome sequencing of Impatiens uliginosa and the analysis of candidate genes related to spur development
Source: BMC Plant Biol. 2022 Dec 1;22:553. doi: 10.1186/s12870-022-03894-1 (PMC9713998; doi:10.1186/s12870-022-03894-1)

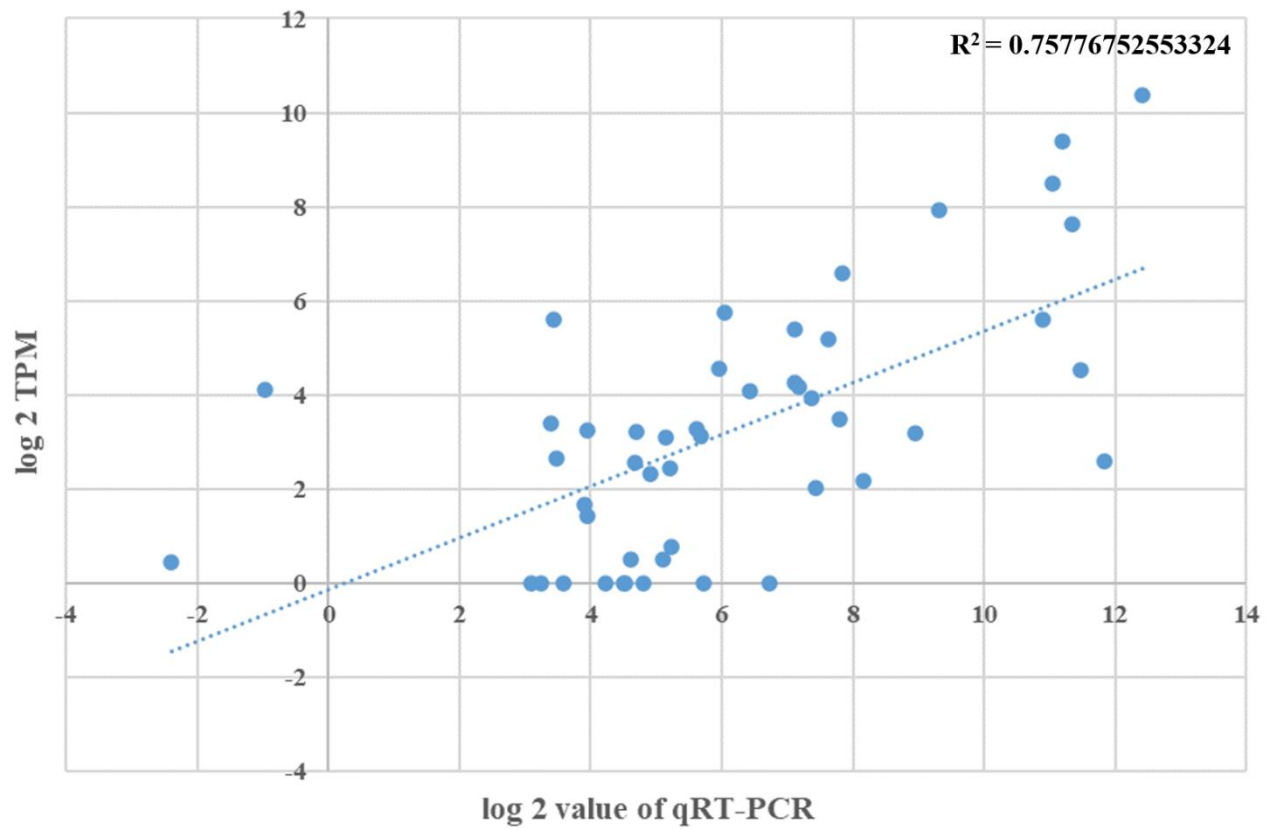

Figure S4 Correlation between RNA-seq expression levels (TPM) and qRT-PCR expression levels.

Supplement: Supplementary file 4 — Additional file 4. [file 12870_2022_3894_MOESM4_ESM.pdf]
