# Supplementary material for: De novo transcriptome sequencing of Impatiens uliginosa and the analysis of candidate genes related to spur development
Source: BMC Plant Biol. 2022 Dec 1;22:553. doi: 10.1186/s12870-022-03894-1 (PMC9713998; doi:10.1186/s12870-022-03894-1)
Supplement: Supplementary file 5 — Additional file 5. [file 12870_2022_3894_MOESM5_ESM.pdf]

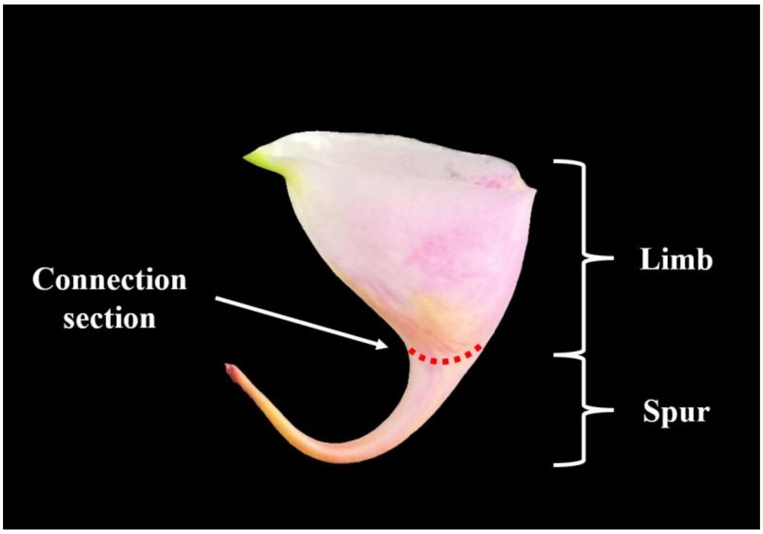

Figure S5 The labellum of *I. uliginosa*. The funnel-shaped part is the limb, and the curved tubular part is the spur, dashed red line indicates the connection section.
